# Multi-Process Remora Enhanced Hyperparameters of Convolutional Neural Network for Lung Cancer Prediction

**DOI:** 10.3390/biomedicines11030679

**Published:** 2023-02-23

**Authors:** Jothi Prabha Appadurai, Suganeshwari G, Balasubramanian Prabhu Kavin, Kavitha C, Wen-Cheng Lai

**Affiliations:** 1 Computer Science and Engineering Department, Kakatiya Institute of Technology and Science, Warangal 506015, Telangana, India; 2 School of Computer Science and Engineering, Vellore Institute of Technology, Chennai 600127, Tamil Nadu, India; 3 Department of Data Science and Business Systems, College of Engineering and Technology, SRM Institute of Science and Technology, SRM Nagar, Chengalpattu District, Chennai 603203, Tamil Nadu, India; 4 Department of Computer Science and Engineering, Sathyabama Institute of Science and Technology, Chennai 600119, Tamil Nadu, India; 5 Bachelor Program in Industrial Projects, National Yunlin University of Science and Technology, Douliu 640301, Taiwan; 6 Department Electronic Engineering, National Yunlin University of Science and Technology, Douliu 640301, Taiwan

**Keywords:** lung cancer prediction, remora optimization algorithm, CT image, performance matrices, feature extraction and pre-processing

## Abstract

In recent years, lung cancer prediction is an essential topic for reducing the death rate of humans. In the literature section, some papers are reviewed that reduce the accuracy level during the prediction stage. Hence, in this paper, we develop a Multi-Process Remora Optimized Hyperparameters of Convolutional Neural Network (MPROH-CNN) aimed at lung cancer prediction. The proposed technique can be utilized to detect the CT images of the human lung. The proposed technique proceeds with four phases, including pre-processing, feature extraction and classification. Initially, the databases are collected from the open-source system. After that, the collected CT images contain unwanted noise, which affects classification efficiency. So, the pre-processing techniques can be considered to remove unwanted noise from the input images, such as filtering and contrast enhancement. Following that, the essential features are extracted with the assistance of feature extraction techniques such as histogram, texture and wavelet. The extracted features are utilized to classification stage. The proposed classifier is a combination of the Remora Optimization Algorithm (ROA) and Convolutional Neural Network (CNN). In the CNN, the ROA is utilized for multi process optimization such as structure optimization and hyperparameter optimization. The proposed methodology is implemented in MATLAB and performances are evaluated by utilized performance matrices such as accuracy, precision, recall, specificity, sensitivity and F_Measure. To validate the projected approach, it is compared with the traditional techniques CNN, CNN-Particle Swarm Optimization (PSO) and CNN-Firefly Algorithm (FA), respectively. From the analysis, the proposed method achieved a 0.98 accuracy level in the lung cancer prediction.

## 1. Introduction

Medical image evaluation can be an unexpected authority in the application of the well-being sector, specifically in clinical examination in addition to non-invasive treatment. Attained restorative images like ultrasound imaging, MRI, CT and X-rays are utilized, aimed at a specific classification. In medial image processing, CT can be a unity of the filtering techniques that utilize striking arenas toward collect images in films [1,2]. Lung cancer is a unity of its severe cancers which leads to 1.61 million deaths per year. In Indonesia, this cancer can be hierarchical in the third position among the ubiquitous cancers for the main portion, initiated in the MIoT middles [3,4,5]. The survival rate is higher if the cancer is identified in the initial phases. The primary identification of lung cancer is not an easy assignment. Mostly, 80% of the affected roles are detected efficiently only at the propelled phase or center of cancer [6,7,8]. 

Cellular dysfunction (cancer) in the lungs ranks subsequent among men and tenth among women worldwide. The data assumed in these statistics are an overall picture of lung cancer in the lung area system with four basic stages [9,10,11]. Lung cancer is the third greatest common malignant development after breast and colon tumors in women. The feature extraction procedure is a unity of the most direct and effective dimension discount procedures in image processing [12]. The unity of the notable features of CT imaging is its insignificant personality. The ascent of the points, in contrast to the equivalent imaging methods, is a visible oddity [13,14,15,16].

A set of selected or extracted features will remove compatible data for the reduction cycle from the information [17,18]. The decreased features can be prepared for a support vector machine and assigned to test. Models are used for lung cancer diagnosis in the lung image sequence, such as binarization image pre-processing with neural network models [19,20]. The current investigation work for lung cancer in the lung line was performed using an 80% accurate neural network model [21]. Different experiments have been conducted on lung cancer in lung characteristics and classifiers; for example, the Support Vector Machine (SVM), k-nearest neighbors (KNN) and Artificial Neural Network (ANN). SVM is a generally effective learning technique in light of measurable learning theory. Nevertheless, these procedures are expensive and recognize cellular dysfunction in the lungs at its highest levels [22,23], owing to the likelihood of tolerance to be exceptionally low. Previous identification of malignant growths is cooperative in fully recovering from the infection. Therefore, the need to develop a process to identify the destructive bubble phenomenon at an early stage expands [24,25].

The main contribution of the research is presented as follows:

❖ In this paper, to develop MPROH-CNN aimed at lung cancer prediction, the proposed technique is utilized to detect the CT images of the human lung;
❖ The proposed technique proceeds with four phases, including pre-processing, feature extraction, and classification. Initially, the databases are collected from the open-source system;
❖ After that, the collected CT images contain unwanted noise, which affects the classification efficiency. So, the pre-processing techniques can be considered to remove unwanted noise from the input images, such as filtering and contrast enhancement;
❖ Following that, the essential features are extracted with the assistance of feature extraction techniques such as histogram, texture and wavelet. The extracted features are utilized in the classification stage.

## 2. Related Works

Different kinds of methods are developed by researchers for lung cancer prediction. A few of these works are reviewed in this section. 

Mesut Togacar et al. [26] have introduced a convolutional neural network (CNN) for classification and extraction requirements. Used toward building the record speed of arrangement, image-enhancing methods such as filling, flat turning, zooming and cutting were used in the database when making the models. Considering the extraordinary effects of the AlexNet model, the elements derived from the final fully connected layer of the design can be used independently as a contribution toward Softmax classifiers, linear regression (LR), linear discriminant analysis (LDA), decision tree (DT), support vector machine (SVM) and k-nearest neighbors (KNN).

Wenqing Sun et al. [27] have introduced a planned feature using multichannel ROI in view of deeply organized calculations for mechanized cellular fracture in lung detection. This study should break down the ability to extract naturally produced features that include in-depth computational calculations in lung bulb CT image analysis, in addition to helping with its computer-aided diagnosis (CADx) utilizing conventional computer hand-made features. Each of the 1018 cases was derived from the Lung Image Database Consortium (LIDC) in the public lung database. The nodules can be separated by the identities of four radiologists, and 134,668 examples were made by pivot for each cut of the nodule images.

Asuntha et al. [28] have provided in-depth information on how to identify malignant lung nodules from the lung image and how to regulate cellular dysfunction in the lungs and determine its severity. To identify the area of dangerous lung nodules, this novel work uses in-depth learning techniques. This work uses advanced element extraction methods such as Zernike Moment, Local Binary Pattern (LBP), Scale Invariant Feature Transform (SIFT), wavelet transform-based features and Histogram of oriented Gradients (HoG). For extracting features, Fuzzy Particle Swarm Optimization (FPSO), volumetric and intensity features and geometric were used to select the best components in the context of removing surface, mathematical, volumetric and energy highlights. For a long time, these features were characterized by the use of in-depth learning.

Kemal Polat et al. [29] have presented an artificial immune recognition system (AIRS) principles component analysis (PCA) and fuzzy weighting pre-processing aimed at lung cancer prediction. This developed method contained three phases. Initially, the lung cancer dimension dataset containing fifty-seven features was decreased to four features with the consideration of principles component analysis. After that, the novel weighting technique related to fuzzy weighting pre-processing was consumed as a pre-processing phase before the projected classifier. At last, the AIRS was utilized to detect lung cancer from the image. 

Mehedi Masud et al. [30] have introduced a machine-learning method to detect malignant growths in the lungs and colon. Malignant growth determination can be robotized using the capabilities of AI, allowing it to be estimated faster than expected and with lower cost. With the assistance of today’s Digital Image Processing (DIP) and Deep Learning (DL) practices, this article writes a characteristic structure that separates the five kinds of lungs in addition to colon tissue (two benign and three malignant) through the breaking down of their histopathological images. The comparison analysis of the existing works are presented in Table 1.

ML calculations have been used to predict and classify different types of biomedical images. Advances in deep learning (DL) computations have empowered machines to handle high-level information such as images, multi-layer body images and video. DL is a subfield of ML that depicts learning calculations enlivened by the design and ability of the human mind. DL uses the power of ANNs to accomplish enhanced design recognition capabilities. Most importantly, it is obvious that AI has provided another aspect to the field of clinical diagnosis, and it is constantly evolving into an appropriate alternative to conventional symptomatic strategies. However, AI is still far from gaining control over the protest area. Although AI models are guaranteed on paper and in controlled trials, they have not yet reached the level of reliability where they can be handed the obligation of pursuing life-changing choices. Of course, a few basic indicator strategies are accomplished exclusively by machines with very few human recommendations. Nevertheless, AI strategies are often inaccurate and their presentation in the practical context is referenced. Furthermore, there are some moral issues. Whatever it is, these stimuli further open the field for experimentation, which is exceptionally welcome for scientists. Moreover, they deal with these difficulties by collecting general knowledge, developing improved learning calculations and placing samples resulting from thorough experiments. In this paper, we aim to illustrate the result of comparable labor. Using another arrangement of histopathological images, we have developed a novel classification technique based on the CNN for the detection of cellular dysfunction in the lungs.

## 3. Proposed Methodology

**Figure 1:**
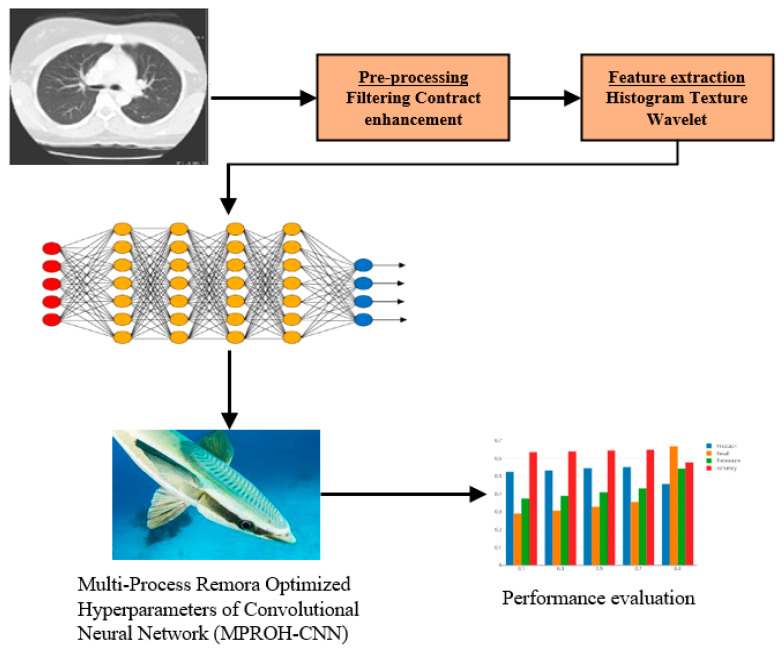
Design of the proposed methodology.

Lung cancer can be a serious disease which develops from the unnecessary growth of cells in lung tissue. The previous identification of cancerous cells is essential in the lungs, as they provide oxygen to the human body in addition to the excretion of carbon dioxide in the human body as an outcome of energetic behaviors. In this paper, we aim to develop the Multi-Process Remora Optimized Hyperparameters of Convolutional Neural Network (MPROH-CNN) for lung cancer prediction. The proposed technique is utilized to identify the CT images of the lung. The proposed technique proceeds with four phases, including pre-processing, feature extraction and classification. Initially, the databases are collected from the open-source system [31,32]. This dataset consists of CT lung cancer images with various classes. From the database, 80% of the data is utilized for training the network, and the remaining 20% of data is utilized for testing the network. After that, the collected CT images contain unwanted noise, which affects the classification efficiency. So, the pre-processing techniques can be considered to reduce unwanted noise from the input images, such as filtering and contrast enhancement. Following that, the essential features are extracted with the assistance of feature extraction techniques such as histogram, texture and wavelet. The extracted features are utilized in the classification stage. The proposed classifier is a combination of the Remora Optimization Algorithm (ROA) and Convolutional Neural Network (CNN). In the CNN, the ROA is utilized for multi-process optimization, such as structure optimization and hyperparameter optimization. The complete design of the projected technique is shown in Figure 1.

Normally, the classification proceeds with two phases: the training phase and the testing phase. The proposed classifier can be trained with the consideration of the selected features. Additionally, during the testing process, the results of the classification process should reveal if the image consists of the lung cancer portion or else normal portions. 

### 3.1. Pre-Processing

The gathered CT lung cancer images contain the unwanted noises which should reduce the empowering of the performance of the projected technique. The noise of the image is reduced with the consumption of the filter. The image contain noise in the final pixels nearby, and pixel worth can be anywhere from 0 s to 255 s by formerly varying the pixel rated with the mean value. Once noise removal from the image is completed, the contrast is enhanced with the assistance of the adaptive histogram equalization technique. The equalization technique is formulated as follows:

(1) $$\begin{array}{cc} CONTRAST\left(I,J\right)=RANK*MAX_{Intensity\left(I,J\right)} & i.e.,\;initially\;rank=0,\;rank=0+1 \end{array}$$

The histogram in the initial location of every line can be achieved using the principal location of the final row through the trailing column, which contains the novel initial row. The complexity of the CT images is improved and set with the period, which it continuously identifies as the image gray level in addition to changing the scattering of two neighboring gray levels in the novel histogram.

### 3.2. Feature Extraction

The aim of the feature extraction method is to denote the image in its unique and compact procedure of matrix vectors or solitary parameters. The feature extraction calculates the dimensionality reduction in the collected images (that are used in the classification). This process is utilized to reduce the input information to a decreased illustrative set of features [33,34]. The features can be used to empower the classification accuracy. To reduce the information through computing the positive characteristics of images, the feature extraction process is utilized. In this research, wavelet features, texture features and histogram features are utilized for extracting the essential features from the CT image.

#### 3.2.1. Wavelet Features

This technique provides image management data due to its advantageous features. The DWT analysis of a direct variation is an operation on the information vector, and this length can be correlated with energy [35]. The wavelet transforms the feature extraction, which can be implemented by the two phases. Initially, the sub band of the original image can be introduced, and this sub band can be validated with the assistance of different resolutions. Wavelet can be an efficient numerical technique to contain extraction and can be utilized to separate the wavelet coefficients, which can be figured out by taking the conventional coarse coefficient, presented as follows:

(2) $$Coefficient\left[A_{T}\right]=\delta_{AT}$$

Here, $\delta_{AT}$ can be represented as a mean parameter of the possible coefficient. At first, the images can be sent to the low pass channel that shades the low recurrence image with the basis of reference recurrence. After that, the image signals can be sent toward the high pass filter that is utilized to reduce the high-frequency beat signals outstanding the reference recurrence. 

#### 3.2.2. Texture Features

This feature can be removed with consideration of the input image. The abnormality of the image can be a blowout in the image, and the textural location of every class can be efficient, which assists in achieving the best classification accuracy. The GLCM is a numerical technique for managing the surface, which explains the spatial validation of pixels. The GLCM operations [36,37,38] are utilized to extract the texture of the image through the computation of the recurrence of the pair of pixels presented with similar parameters. Normally, this feature can be computed with the consideration of utilizing GLCM probability parameters. In addition, it contains anywhere in the period of 22 features between that some features can be well-thought-out for the presented research related to CT lung image detection procedure:

(3) $$GP_{IJ}=\frac{F_{IJ}}{\sum_{I,J=0}^{L-1}F_{IJ}}$$

Based on the above formulation, I and J can be described as the displacement vector with the specific window size, L can be described as the quantized gray levels count and $F_{IJ}$ can be described as the frequency of occurrences between two gray levels.

Correlations:

The correlation function computes the stable variance of the gray levels of combining pixels. The input image correlation trailing represents an efficient process that misuses tracking, and image registration can be enabled for the computation of changes in images. 

Contrast:

This contrast function is utilized to compute the spatial recurrence of the image in addition to changing the moments of GLCM. This function is a variance among the reference parameter and the maximum parameters of a neighboring arrangement of pixels.

Homogeneity:

This function can be normally defined as the contrast minute that computes the image homogeneity consuming the prevalent parameters aimed at slight gray tone variations in a couple of parameters. Combined with these lines, this function can compute behaviors in prevalent parameters for small contrast images.

Entropy:

This function is defined as the quantity of information in the image that can be necessarily aimed at the compression procedure. The image with small entropy empowers large runs and tiny contrasts of pixels in the image with specified parameters.

Energy:

This energy function is a sporadic constancy in gray level distribution, and the highest constant parameters will form the highest vitality of the unit.

#### 3.2.3. Histogram Features

In this feature, the image can be defined as the name of pixels. This histogram is defined as the number of pixels in an image during every power parameter. Changing the power parameters of the image histogram [39,40] which is about competitions a defined histogram. Based on the input image, a complete parameter of gray levels can be computed with the basis of the histogram technique. Here, 256 gray levels are considered that vary between 0 and 255. This function consists of communal features such as standard, kurtosis, skewness, mean and variance.

##### Kurtosis

This is defined as the metric of the possible variation of the reference parameter random variable anomaly of the image. Skewness and kurtosis can be utilized in the statistical validation to achieve a vision into a distribution shape.

##### Skewness

The skewness can be computed related to the histogram tail. The histogram end parameter is divided into two pairs: negative and positive.

##### Standard Deviation

This is a measure that can be a square root of the alteration mentioning image contrast. The image contrast stage can be computed through low and high variance parameters. This function defines a huge contrast image which contains a huge alteration while a low variance is a low contrast image.

##### Mean

The mean provides the average gray level of every period and assists only as harsh information of power, not by any stretch of the texture behaviors of the image.

##### Variance

The variance provides the count of gray level variations after the mean parameter of the gray level parameter. The arithmetical variation, which is the length of line alteration of a specific period, can be considered to identify little profile contrasts in the texture.

### 3.3. Multi-Process Convolutional Neural Network

**Figure 2:**
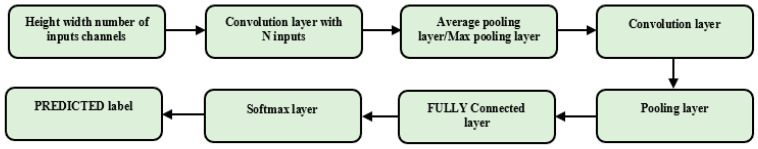
CNN architecture.

The initial efficient multi-layer NN design or deep structure of CNN is illustrated in Figure 2. The CNN is merged from the theory of the sub-region of open arenas developed through the authors. The CNN decreases the complexity of the conventional neural systems as multi-layer perceptron (MLP) by sharing filters aimed at the whole image. The dimension of the kernel matrix or filter can be huge or lesser when compared with the input image and can decrease the necessity of the count variables correlated with every pair of connections between output and input. This structure chains the characteristics of equivariance with sparse influences, which additionally empower the output change in a similar path as the input. The most efficient objective of CNN is the necessity of less variables as contrasted to the remaining conventional NNs [41]. This process decreases the computation complexity and memory necessary, which enhances the performance. This is mainly designed with three layers, such as a fully connected layer, pooling layer and convolutional layer.

The implementation CNN parameters are listed in Table 2.

In the CNN, the convolutional layer is utilized to extract the features and learns filters with the consideration of the backpropagation technique. Every filter attaches the output to specific inputs that overlap in the required arenas of the last layer. This filter consists of bias in addition to weight variables toward the train, and this variable can be shared through multiple locations. The spatial characteristics associated with neighboring pixels assists in the training procedure. The convolution process empowers the layer to identify similar objects in multiple images with various positions. After that, the polling layer is utilized to decrease input dimension, which manages translation invariance toward feature transformation, empowers to achieve robustness and avoids overfitting. The max pooling layer can contain convolution layers toward save features, identified wherever, and can be operated autonomously and aimed at every channel. This progresses toward achieving the best invariance and robustness. An additional technique is regular polling, which can also be utilized, but max polling can be utilized normally because of its faster convergence characteristics. Some other related techniques are stochastic pooling, aimed at best generalization; spatial pyramid polling, aimed at enhancing the detection accuracy of images of arbitrary sized and def-pooling, and at training from the distortion of images. The fully connected layer can be connected after the final pooling layer. The initial fully connected layer decreases the measurement of the feature vector achieved from the last layer to a single dimensional vector. It can be thickly linked with remaining layers and needs different variables with an improvement in the exertions of processing time. The last fully connected layer can be designed through the classification layer or regression layer.

### 3.4. Remora Optimization Algorithm

The remora is well known for its aptitude to swim with whales or additional sea creatures or marine bodies in pursuit of saving energy and seeking safety from predators. It is usually transported in tropical water. However, it additionally follows like a virus into its host’s water. The remora primarily benefits from other fish or invertebrates. Once it comes to a section of the ocean rich in food, it leaves the host, eats the food and then latches back onto a host via suction [42] to continue moving to new hosts and other sea areas. The initialization of the ROA algorithm is a present position with the number of remora and different dimensions in the search space. The initialization process is presented as follows

(4) $$r_{I}=\left(r_{I1},r_{I2},\ldots ,r_{ID}\right)$$

where D is defined as the dimension of the remora and I is defined as the number of remoras.

*Exploration stage with free travel*

*SFO technique*

The remora is attached to the swordfish, and this location is defined as the updating process with a similar period. This process is formulated as follows:

(5) $$r_{I}^{T+1}=r_{best}^{T}\left[RAND\left(0,1\right)*\left(\frac{r_{best}^{T}+r_{RAND}^{T}}{2}\right)-r_{RAND}^{T}\right]$$

where $r_{RAND}$ is defined as the random location, T can be described as the maximum number of iterations and t is defined as the present iterations. The elite choose remora historical optimal solution which proceeds the updating process. Algorithm 1 is the pseudocode representation of Remora Optimization Algorithm.
**Algorithm 1:** Pseudocode of Remora Optimization AlgorithmInitialize the random location with their population and memory location Initialize the best solution in addition related with optimal fitness While T>Tmax  Calculate the fitness function parameter of every remora  Validate if any search agent goes beyond the search location  Update α,B,V
  For every remora indexed by I do If *H*$\left(I\right)=0$ then  Based on Equation (7), the position of attached whales is updated Else if $H\left(I\right)=1$ then  Based on Equation (5), the position of attached sailfishes is updated End if  Compute the one-step identification by (6)  Compute the parameter $H\left(I\right)$ through Equations (7) and (8) to validate if host change is required  If the host is not changed, Equation (12) can be utilized as the host feeding mode for remora End for End while

*Experience Attack*

To compute the required change of host, the experience attack is considered. The experience attack is formulated as follows:

(6) $$r_{att}=r_{I}^{T}+\left(r_{I}^{T}-r_{pre}\right)*randn$$

Here, $r_{att}$ is defined as tentative step and $r_{pre}$ is defined as the previous generation position. 

Exploitation (eat thoughtfully)

*WOA method*

In the foundation of the normal WOA technique, the updating of position formulation is presented as follows:

(7) $$r_{I+1}=d*E^{a}*cos\left(2\pi \alpha \right)+r_{I}$$

(8) $$\alpha =RAND\left(0,1\right)*\left(a-1\right)+1$$

(9) $$a=-\left[1+\frac{T}{t}\right]$$

(10) $$d=\left|r_{best}-r_{I}\right|$$

In a higher solution space, the remora can be a whale, and its positions are decreased similarly. Here, α is defined as the random number in [−1, 1] and d is defined as distance between prey and hunter. Additionally, the random number is presented [−2, −1].

*Host feeding*

This is an exploitation procedure. In this phase, the solution space is decreased in the host location space. The crowd moving on the small steps is formulated as follows:

(11) $$r_{I}^{T}=r_{I}^{T}+A$$

(12) $$A=B*\left(r_{I}^{T}-c*r_{best}\right)\;$$

(13) $$B=2*V*RAND\left(0,1\right)-V$$

(14) $$V=2*\left(1-\frac{T}{Maximum\;iteration}\right)$$

Here, C is defined as the remora position and A is defined as minor steps of movement that can be correlated toward the host volume space [43].

### 3.5. The Proposed Methodology: MROA-CNN

**Figure 3:**
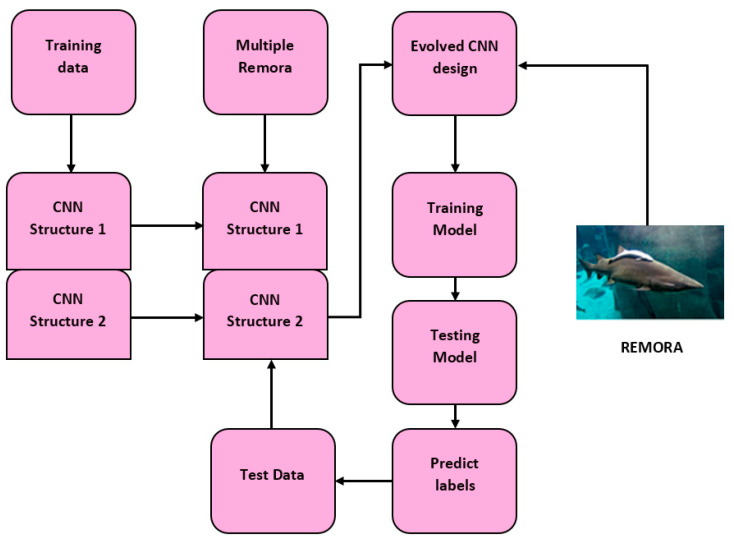
Process of the proposed technique.

The training of CNN proceeding with the basis of a backpropagation technique consists of two major processes: The initial process is a feed forward process in that specific features can be achieved by providing different filters at every layer. This feature passes by various layers in a forward way, and the last layer calculates the specific output. In the next process, the error parameter is a variance between the present output and the predictable output, which can be calculated and then backpropagated toward the last layers aimed at the optimal change of variables by utilizing a gradient descent approach [44]. Different regularization techniques can be utilized to empower generalization ability and decrease overfitting. This portion defined the general foundation of the hybrid technique, detailed architecture, algorithms and flow diagram. The block diagram of the projected approach is presented in Figure 3. 

The MROA utilized many remoras to compute the design of CNN in addition to hyper parameters. Every remora defines the possible design of CNN. The last layer of CNN is a Softmax layer to calculate the occurrence probability of every class. The achieved accuracy defines the fitness parameter of every remora. ROA normally optimizes the hyperparameters of a CNN. In addition, it lastly meets a shape with the optimal fitness parameter. The designed CNN structure with the best set of hyperparameters is qualified with the consumption of a huge number of training numbers. The selected CNN with trained variable values can be utilized additionally for the classification of unidentified examples. In this CNN, the sigmoid function is utilized as the inertial weight for calculating velocity, given below:

(15) $$\omega \left(t\right)=\left\{\begin{array}{c} 0.9\;when\;T<AT_{max}\\ \frac{1}{1+e^{\left(10T-\frac{2}{max}\right)/}}\;otherwise \end{array}\right.$$

#### 3.5.1. Phase 1: Hyperparameters Search Space with Remora Initialization

The maximum and minimum values of CNN hyperparameters manage the sizes of the remora in the specified search space. A specific variety of hyperparameters can be utilized, aimed at complete standard databases in the implementation of validation. The multi-level ROA enhances the 11 hyperparameters of a CNN. The initial stage of the remora contains three hyperparameters in a remora, such as a count of fully connected layers (nf), count of polling layers (np) and count of convolution layers (nc). Additionally, the next process of remora consists of eight parameters, such as the count of output neurons in a fully connected layer (op), padding pixels in a polling layer (p-pp), max pooling layer with a filter size (p-fs), convolutional layer stride side (c-ss), convolutional layer padding requirements (c-pp), convolutional layer kernel and filter size (c-fs) and convolutional layer number of filters (c-nf). The remoras are initialized in the specific period to compute the hyperparameters of the optimal set in the CNN.

#### 3.5.2. Phase 2: ROA Algorithm Parameter Values

The updated position vector dimension of the remora can be selected as the integer parameter nearest to the lower in the period. The remora at the level 1 process contains five remora, and every remora can be the size of vector 3. So, the dimension of the remora in the stage is 5 × 3. The initial parameter defines the count of convolutional layers. Additionally, the next level also considers the convolutional layer parameters. The initial weight of the network is selected with the consideration of Equation (4).

#### 3.5.3. Phase 3: Remora Structure

In the remora, it contains the five remora, and each defines the configuration of the CNN. Every remora can be lengthy at stage 2 with the population of a likely hyperparameter set of a CNN which converges to the evolution finish. Five remoras can be proceeded at level 2, and five more remoras will be created at level 2. These eight parameters that are required to be selected are the fully connected layer with number of output neurons, pooling layer padding bit, stride size, filter size, convolutional layer stride size, padding bits, filter size and number of filters. The proposed methodology repeats, computing the optimal CNN shape in a search space.

#### 3.5.4. Phase 4: Fitness Computation

In the projected algorithm, CNN proceeding with a Softmax layer computes the fitness of every remora. The hyperparameter set of CNN provides the best accuracy. The fitness function of the algorithm is presented as follows:

(16) $$FF\left(S_{IJ}\right)=CNN\left(S_{I},S_{IJ}\right)$$

where

(17) $$S_{I}=\left(nc,np,nf\right),S_{IJ}=\left(op,p-pp,p-fs,c-ss,c-pp,c-fs,c-nf\right)$$

Based on the fitness function, the ROA proceeds and optimal best parameters are selected, which are sent to the CNN classifier for lung cancer prediction.

## 4. Experiments and Results

The proposed methodology is implemented in MATLAB, and performances are evaluated by using performance matrices such as accuracy, precision, recall, specificity, sensitivity and F_Measure. To validate the projected approach, it is compared with conventional techniques, such as CNN, CNN-PSO and CNN-FA, respectively. The databases are collected from the open-source system. The databases contain the four different classes: Adenocarcinoma, Large cell carcinoma, Normal and Squamous cell carcinoma. The different class images are utilized to train the network and test the network for a diagnosis of lung cancer. From the databases, 80% of the images are utilized for training the network and the remaining 20% of images are utilized for testing the network. Similarly, the data and design parameters are considered in the conventional techniques for comparison performance. The simulation variables are presented in Table 3. To validate the proposed method, a two- fold validation is considered. The outcome of the images is presented in Table 4. 

**Figure 4:**
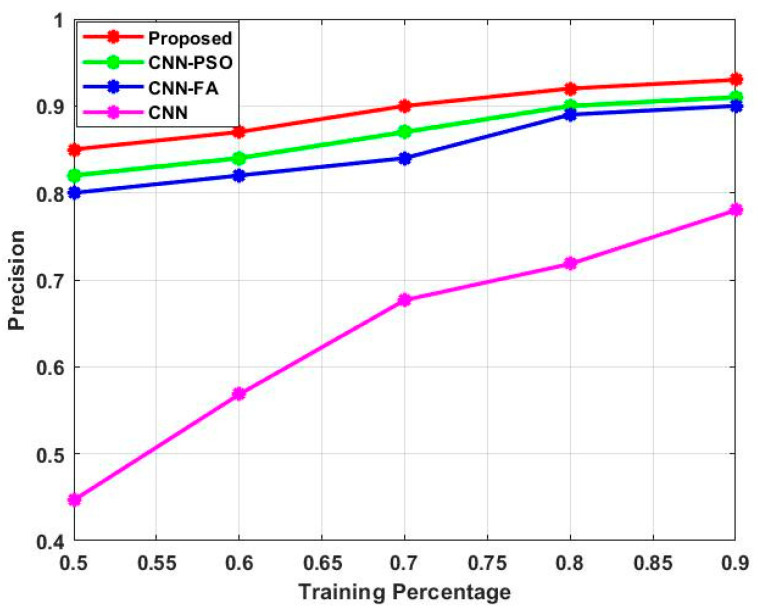
Precision.

**Figure 5:**
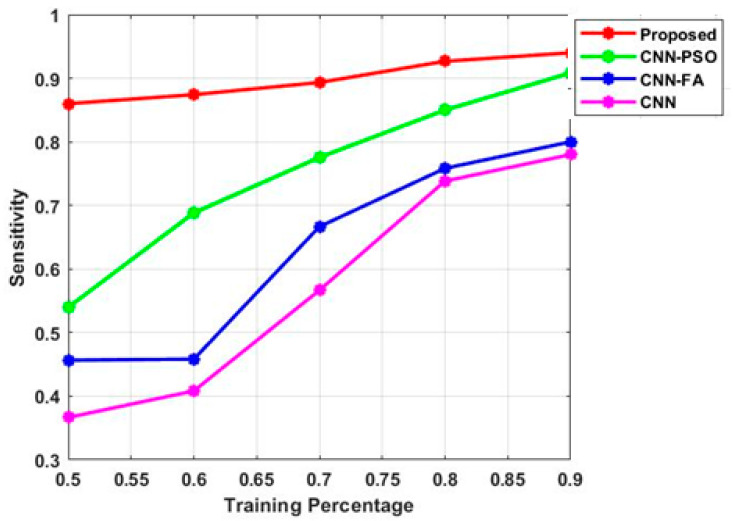
Sensitivity.

The projected technique is validated with the consideration of performance metric precision, illustrated in Figure 4. The projected approach is contrasted with conventional methods, such as CNN-FA, CNN-PSO and CNN. The precision measure of the proposed method is 0.88 during 0.5 training percentage. Similarly, the CNN-PSO, CNN-FA and CNN achieved 0.82, 0.8 and 0.58 at 0.5 training percentage, respectively. The precision measure of the proposed method is 0.91 during 0.8 training percentage. Similarly, the CNN-PSO, CNN-FA and CNN achieved 0.89, 0.85 and 0.77 at 0.8 training percentage, respectively. Related to the analysis, the projected approach attained the efficient outcomes in the measurement of precision. The projected approach is validated with the consideration of performance metric sensitivity, which is illustrated in Figure 5. The projected technique is contrasted with conventional methods, such as CNN-FA, CNN-PSO and CNN. The sensitivity measure of the proposed method is 0.89 during 0.5 training percentage. Similarly, the CNN-PSO, CNN-FA and CNN achieved 0.52, 0.48 and 0.38 at 0.5 training percentage. The sensitivity measure of the proposed method is 0.92 during 0.8 training percentage. Similarly, the CNN-PSO, CNN-FA and CNN achieved 0.91, 0.78 and 0.72 at 0.8 training percentage. Related to the analysis, the projected approach attains the efficient outcomes in the measurement of sensitivity.

**Figure 6:**
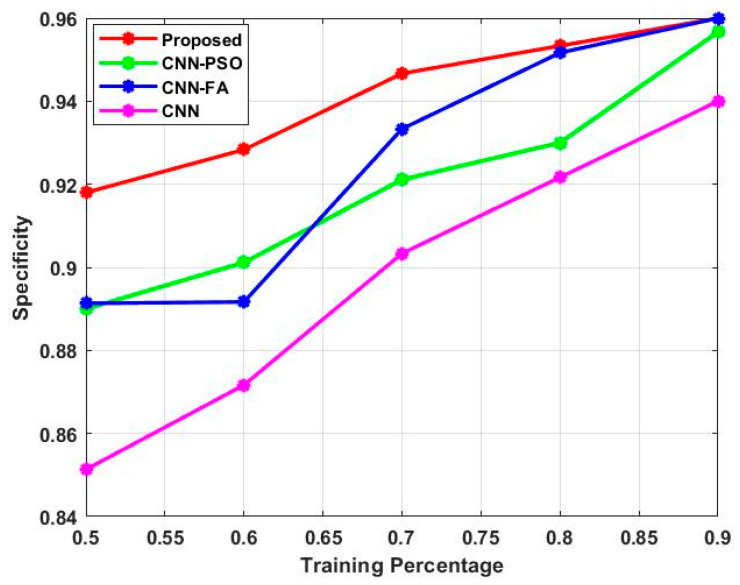
Specificity.

**Figure 7:**
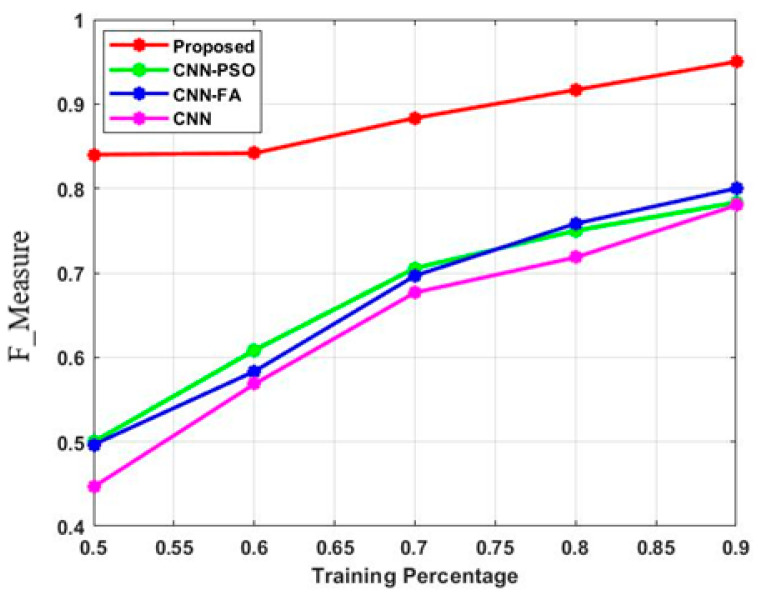
F_Measure.

The projected technique is validated with the consideration of performance metric specificity, which is illustrated in Figure 6. The projected method is contrasted with conventional methods, such as CNN-FA, CNN-PSO and CNN. The specificity measure of the proposed method is 0.92 during 0.5 training percentage. Similarly, the CNN-PSO, CNN-FA and CNN achieved 0.89, 0.89 and 0.85 at 0.5 training percentage. The specificity measure of the proposed method is 0.93 during 0.8 training percentage. Similarly, the CNN-PSO, CNN-FA and CNN achieved 0.91, 0.88 and 0.81 at 0.8 training percentage. Related to the analysis, the projected approach attained efficient outcomes in the measurement of specificity. The projected technique is validated with the consideration of performance metric F_Measure, which is illustrated in Figure 7. The projected method is contrasted with conventional methods, such as CNN-FA, CNN-PSO and CNN. The F_Measure of the proposed method is 0.83 during 0.5 training percentage. Similarly, the CNN-PSO, CNN-FA and CNN achieved 0.51, 0.51 and 0.45 at 0.5 training percentage. The F_Measure of the proposed method is 0.86 during 0.8 training percentage. Similarly, the CNN-PSO, CNN-FA and CNN achieved 0.82, 0.78 and 0.67 at 0.8 training percentage. Related to the analysis, the F_Measure approach attained efficient outcomes in the measurement of F_Measure.

**Figure 8:**
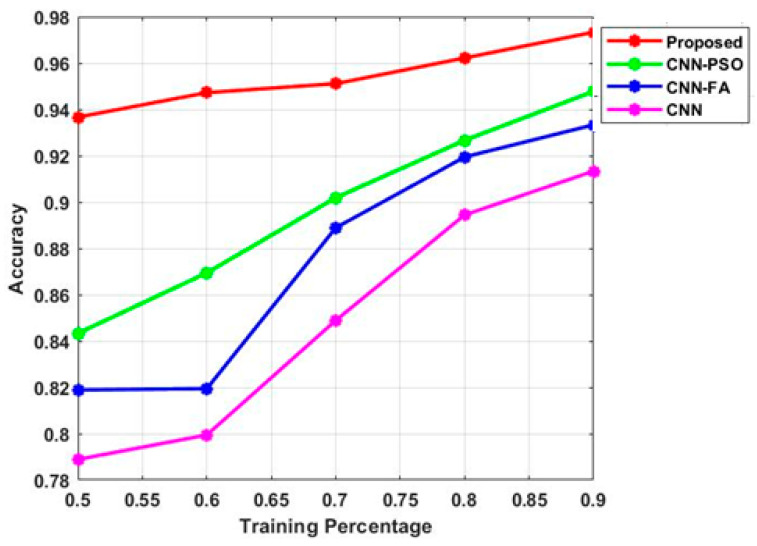
Accuracy.

**Figure 9:**
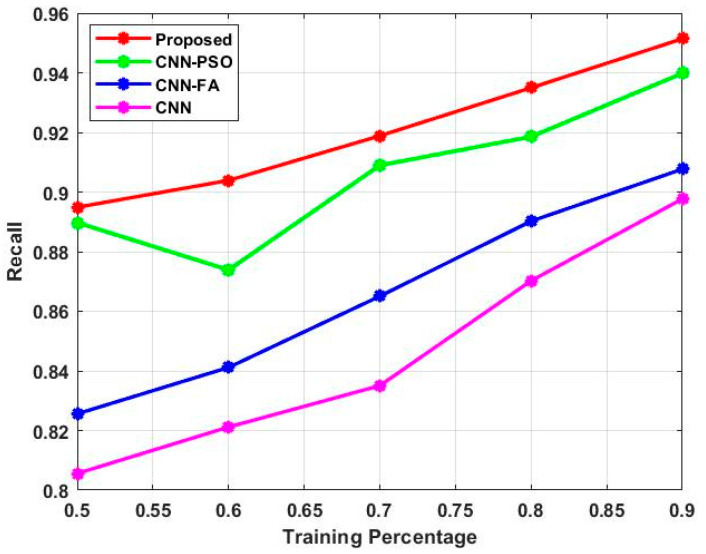
Recall.

**Figure 10:**
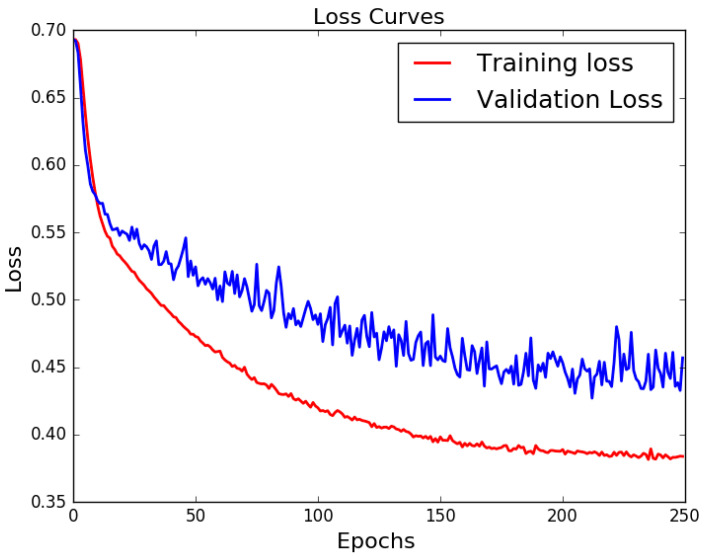
Overfitting and underfitting curves.

The projected technique is validated with the consideration of performance metric accuracy, which is illustrated in Figure 8. The projected method is contrasted with conventional methods, such as CNN-FA, CNN-PSO and CNN. The accuracy measure of the proposed method is 0.94 during 0.5 training percentage. Similarly, the CNN-PSO, CNN-FA and CNN achieved 0.85, 0.82 and 0.79 at 0.5 training percentage. The accuracy measure of the proposed method is 0.91 during 0.8 training percentage. Similarly, the CNN-PSO, CNN-FA and CNN achieved 0.85, 0.81 and 0.79 at 0.8 training percentage. Related to the analysis, the accuracy approach attained efficient outcomes in the measurement of accuracy. The projected technique is validated with the consideration of performance metric recall, which is illustrated in Figure 9. The projected method is contrasted with conventional methods, such as CNN-FA, CNN-PSO and CNN. The recall measure of the proposed method is 0.899 during 0.5 training percentage. Similarly, the CNN-PSO, CNN-FA and CNN achieved 0.891, 0.821 and 0.81 at 0.5 training percentage. The recall measure of the proposed method is 0.879 during 0.8 training percentage. Similarly, the CNN-PSO, CNN-FA and CNN achieved 0.8451, 0.8356 and 0.768 at 0.8 training percentage. Related to the analysis, the recall approach attained efficient outcomes in the measurement of accuracy. The overfitting and underfitting curves of the proposed method are illustrated in Figure 10.

## 5. Conclusions

In this paper, to develop MPROH-CNN for lung cancer prediction, the proposed technique utilized to detect the CT images of the human lung proceeds with four phases, including pre-processing, feature extraction and classification. Initially, the databases are collected from the open-source system. After that, the collected CT images contain unwanted noise which affects the classification efficiency. So, the pre-processing techniques can be considered to eliminate unwanted noise from the input images, such as filtering and contrast enhancement. After that, the essential features were extracted with the assistance of feature extraction techniques, such as histogram, texture and wavelet. The extracted features were utilized in the classification stage. The proposed classifier is a combination of ROA and CNN. In the CNN, the ROA is utilized for multi-process optimization, such as structure optimization and hyperparameter optimization. The proposed methodology has been implemented in MATLAB, and performances have been evaluated by using performance matrices like accuracy, precision, recall, specificity, sensitivity and F_Measure. To validate the projected approach, it has been compared with the conventional techniques, such as CNN, CNN-PSO and CNN-FA, respectively. The proposed technique achieves the precision, sensitivity, specificity, F_Measure, recall and accuracy of 0.88, 0.89, 0.92, 0.83, 0.94 and 0.899, respectively. From the analysis, the projected technique achieved efficient outcomes in the measure of statistical measurements. The proposed method consumes a huge amount of time when utilizing large datasets. To overcome the drawbacks, a new efficient method will be considered to empower the progress of lung cancer detection. 

## Figures and Tables

**Table 1:** Comparison analysis.

References	Methods	Dataset	Accuracy
[26]	Convolutional neural network		95
[27]	Multichannel ROI	LIDC	95
[28]	Fuzzy particle swarm optimization	lung cancer data	96
[29]	Artificial immune recognition system	AIRS	85
[30]	Machine learning	lung colon disease	82
[31]	Proposed method		98

**Table 2:** Implementation variables.

Hyperparameter	Layer	Range
Number of neurons (op)	Fully connected	1
Padding pixels (p-pp)	Pooling layer	0–1
Stride size (p-ss)		1–5
Filter size (p-fs)		1–13
Stride size (c-ss)	Convolution	1–5
Padding pixels (c-pp)		0–1
Filter size (c-fs)		1–13
Number of filters (c-nf)		1–64
Count of fully connected layers (nf)	Fully connected layer	1–5
Count of pooling layers (np)	Number of pooling layers	1–5
Count of convolutional layers (nc)	Number of convolutions	1–5

**Table 3:** Simulation variables.

S. No	Methods	Description	Parameters
1	Remora optimization algorithm	Number of iterations	100
2		Number of search agents	50
3		Lower bound	−10
4		Upper bound	10
5		Epoch	50
6	Particle swarm optimization	Number of particles	100
7		Inertia weight factor	0.9
8		Acceleration constant C1	1.2
9		Acceleration constant C2	1.2
10	Firefly algorithm	Number of fireflies	5
11		Maximum generation	100
12		Alpha (randomness)	0.5
13		Beta min (initial attractiveness)	0.2
14		Gamma (absorption coefficient)	0.5

**Table 4:** Filtered image’, ‘Contrast enhanced image’.

Class	Input	Filter	Contrast
Adenocarcinoma	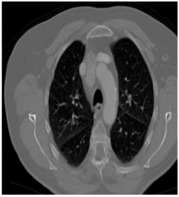	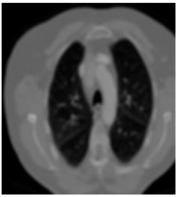	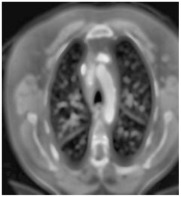
Large cell carcinoma	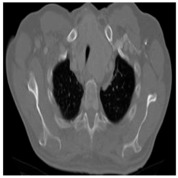	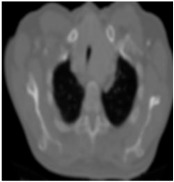	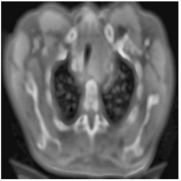
Normal	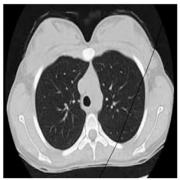	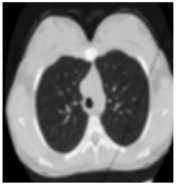	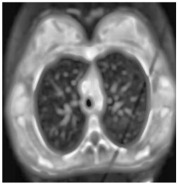
Squamous cell carcinoma	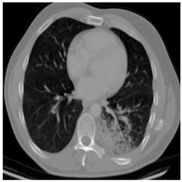	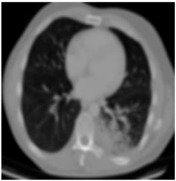	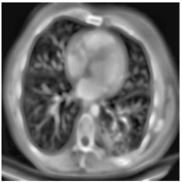

## Data Availability

The datasets used and/or analyzed during the current study are available from the corresponding author on reasonable request.
